# Editorial: Cancer immunity and radiotherapy

**DOI:** 10.3389/fimmu.2026.1950844

**Published:** 2026-08-06

**Authors:** Yaqi Cai, Ruiqi Liu, Haibo Zhang, Yunbei Yang

**Affiliations:** 1 Cancer Center, Department of Radiation Oncology, Zhejiang Provincial People’s Hospital (Affiliated People’s Hospital), Hangzhou Medical College, Hangzhou, Zhejiang, China; 2 Urology and Nephrology Center, Department of Urology, Zhejiang Provincial People’s Hospital (Affiliated People’s Hospital), Hangzhou Medical College, Hangzhou, Zhejiang, China

**Keywords:** abscopal effect, biomarkers, cancer immunotherapy, immune checkpoint inhibitors, immunogenic cell death, radiation-induced toxicity, radiotherapy, tumor microenvironment

## Introduction

Over the past decade, cancer immunotherapy—most prominently immune checkpoint blockade—has transformed the treatment of many malignancies (1). Yet as a standalone modality, it still delivers durable benefit to only a minority of patients, prompting interest in strategies that can broaden and sustain antitumor responses. Radiotherapy, a mainstay of cancer treatment, has emerged as a particularly attractive partner because, beyond its direct cytotoxic effects, it can reshape the tumor microenvironment and potentially enhance antitumor immunity (2).

**Figure 1:**
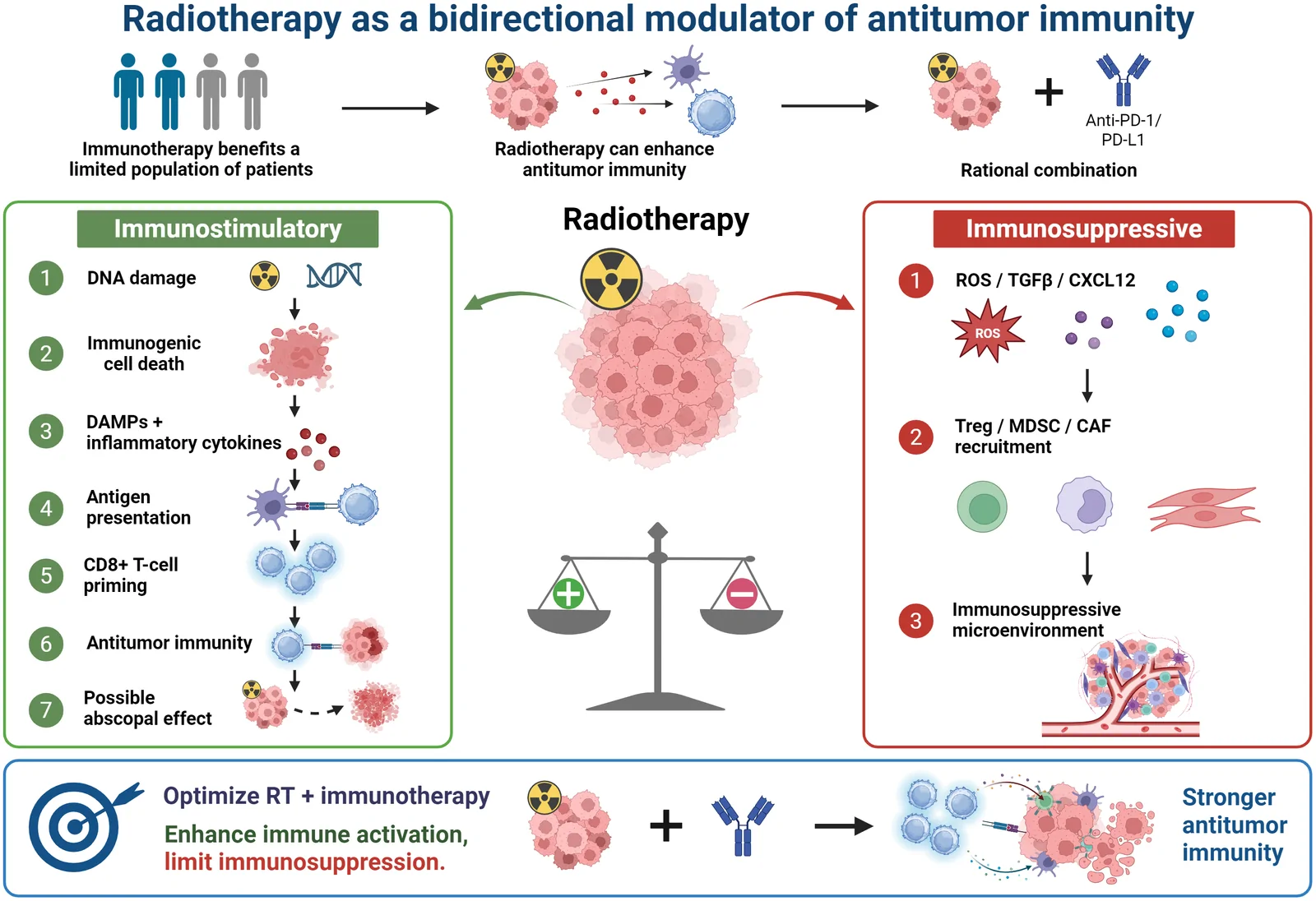
Bidirectional immunomodulatory effects of radiotherapy on antitumor immunity. Radiation can trigger immunostimulatory events including immunogenic cell death, antigen presentation and CD8⁺ T cell priming, but simultaneously induces the production of ROS, TGFβ and CXCL12 to recruit immunosuppressive cell populations and foster a suppressive tumor microenvironment. Optimized combination of radiotherapy with anti-PD-1/PD-L1 blockade enhances immune activation and limits immunosuppression to achieve robust antitumor immunity.

However, radiotherapy has complex and sometimes opposing effects on antitumor immunity. On the one hand, radiation-induced DNA damage triggers immunogenic cell death. This process activates inflammatory cytokine signaling and releases damage-associated molecular patterns (DAMPs) (3). DAMPs enhance antigen presentation and prime cytotoxic T cells (4, 5). These effects can occasionally extend beyond the irradiated field (6). On the other hand, irradiation also generates reactive oxygen species, TGFβ, CXCL12, and related factors (7, 8). These factors recruit regulatory T cells, myeloid-derived suppressor cells, and cancer-associated fibroblasts. Their recruitment reinforces an immunosuppressive microenvironment (9, 10). A better understanding of the molecular determinants that influence this balance is central to improving patient outcomes. Strategies that enhance the immunostimulatory effects of radiotherapy while limiting its immunosuppressive consequences will be critical to optimizing its combination with immunotherapy (**Figure 1**).

This Research Topic, “Cancer Immunity and Radiotherapy,” assembles 16 articles. These include multi-institutional cohort studies, translational analyses, focused reviews, and case reports. Together, they examine the interactions between radiation and the immune system across tumor types. The contributions address the mechanistic determinants of radiation-induced immunity and the clinical integration of radiotherapy with immune checkpoint blockade. They also cover the refinement of radiation delivery, the search for predictive biomarkers, and the management of toxicity and paradoxical responses.

## Radiation as a modulator of antitumor immunity

Radiation reshapes antitumor immunity in part through its effects on the tumor microenvironment. In a wide-ranging review, Luo et al. examine the context-dependent roles of neutrophil extracellular traps (NETs) in the irradiated tumor microenvironment. They describe how radiation drives NET formation through HMGB1–TLR4 signaling, ROS, and autocrine IL-8. The resulting webs can form physical barriers. These barriers exclude CD8+ T cells, shield circulating tumor cells, and promote radio resistance. In other contexts, radiation-recruited N1 neutrophils and NETs reinforce antitumor immunity. The authors propose a microbiota–metabolite–NET–radiation axis. They also discuss targeting strategies (PAD4, HMGB1, and CXCR1/2 inhibitors or DNase I combined with PD-1/PD-L1 blockade). Together, these observations frame radiation as a context-dependent immune modulator rather than a uniformly immunogenic stimulus.

The immune-stimulatory potential of radiotherapy is clinically illustrated by Shu et al. They report on a patient with refractory, TKI-resistant HBV-related metastatic hepatocellular carcinoma (HCC). The patient received third-line treatment with stereotactic body radiotherapy (SBRT) plus a PD-1 inhibitor. Multiple nonirradiated lymph node lesions and portal vein tumor thrombi regressed. This was a clear example of the abscopal effect. AFP levels also declined. Progression-free survival was 10 months, and overall survival was 21 months. These findings illustrate how local radiation may enhance systemic responses to checkpoint blockade beyond the irradiated field.

## Clinical integration of radiotherapy with immune checkpoint blockade

Several contributions extend the evidence from individual case reports to cohort studies. Zhai et al. present a real-world series of 108 patients with metastatic or recurrent HCC. The patients received radiotherapy plus PD-1/PD-L1 inhibitors. The objective response rate was 75% (mRECIST). The 2-year in-field control was 96.7%, and median overall survival was 17 months. The cohort was enriched for poor-prognosis features such as portal vein tumor thrombus. These results motivated a prospective randomized trial.

The remaining reports examine the optimal sequencing of combination therapy and the role of salvage treatment. Fu et al. report findings from a three-institution cohort of unresectable stage III non-small cell lung cancer (NSCLC). They found that induction immunochemotherapy before definitive chemoradiotherapy and consolidation immunotherapy improved overall survival (HR = 0.50). It also reduced distant metastasis. However, it was associated with a higher incidence of grade 3–5 pneumonitis. Jiang et al. describe a patient with limited-stage small-cell lung cancer. The patient developed isolated out-of-field nodal progression during consolidation immunotherapy. Salvage re-irradiation plus continued immunotherapy resulted in durable complete remission. This case suggests that out-of-field relapse does not necessarily indicate treatment failure. Xu et al. combined whole-brain and sequential integrated-boost radiotherapy with sintilimab and bevacizumab in a patient with cervical small-cell carcinoma and more than ten brain metastases. The patient survived for more than three years. Xu et al. also documented a durable response to the PD-L1 inhibitor socazolimab in recurrent, previously irradiated metastatic cervical cancer. Collectively, these reports provide practical guidance on integrating radiation and checkpoint blockade across diverse and challenging clinical settings. This guidance also covers combinations with antiangiogenic partners.

## Optimization of radiotherapy delivery

Radiotherapy fractionation and treatment planning influence both immunological effects and clinical outcomes. Yang et al. retrospectively compared hypofractionated and conventionally fractionated radiotherapy in stage III NSCLC with low tumor mutational burden. This subgroup is typically resistant to conventional radiation. Hypofractionation significantly prolonged median progression-free survival (13 vs. 10 months). Toxicity was comparable. Liang et al. highlight an important consideration for treatment planning. The esophageal tumor shifted by approximately 4.2 cm between fractions during neoadjuvant chemo-immunotherapy. This shift far exceeded conventional planning margins. It underscores the need for frequent image guidance and adaptive replanning when combination therapy rapidly alters tumor anatomy.

## Biomarkers and patient selection

The effective implementation of radio-immunotherapy requires identifying patients who are most likely to benefit. Lai et al. derived a two-gene stemness signature (EMILIN1, CYP4Z1). The signature stratifies breast cancers by radiosensitivity. Radiosensitive tumors were linked to active DNA repair pathways, higher immune checkpoint expression, and lower TIDE scores. These features indicate potential immunotherapy benefit. Yin et al. show that a nomogram predicts response noninvasively in locally advanced and advanced lung cancer. The nomogram was built from peripheral blood lymphocyte subsets. These included natural killer cells, the CD4+/CD8+ ratio, and B cells. Wang et al. examine the potential for treatment de-escalation. They studied locally advanced rectal cancer with deficient mismatch repair (dMMR/MSI-H). Surgery alone was associated with comparable or superior survival relative to added chemo(radio)therapy. This finding identifies an immunologically defined subgroup for whom radiation may be safely omitted. Wang et al. also review molecular imaging of the tumor immune microenvironment. They catalog PET, MRI, and optical probes. These probes visualize T cells, macrophages, and cancer-associated fibroblasts. They can monitor response and guide personalized decisions.

## Toxicity, atypical responses, and emerging local treatment strategies

Combination therapy is also associated with distinct risks. Wang et al. built and validated a nomogram incorporating radiomic and dosimetric parameters (AUC 0.92/0.83). The nomogram predicts radiation esophagitis in NSCLC patients receiving sequential immunotherapy and radiotherapy. It implicates the maximum and mean esophageal doses and a radiomics score. The authors also invoke cGAS–STING-driven inflammation of normal tissue. Zhou et al. report delayed hyperprogressive disease. It emerged five months after immunotherapy, the longest interval documented. Lesion growth after radiation was later controlled by transarterial chemoembolization. This case illustrates that responses to radio-immunotherapy can be both unfavorable and unpredictable. Maswikiti et al. extend the discussion beyond ionizing radiation. They describe a chemotherapy-free regimen for a patient with rare recurrent angiosarcoma involving the scalp and face. The regimen combines photodynamic therapy, targeted therapy, and immunotherapy. The report suggests that other immunogenic, tissue-sparing local modalities may complement or provide alternatives to radiation.

## Conclusion

Collectively, these 16 contributions show that radiotherapy is not merely a cytotoxic adjunct to immunotherapy. It is also an active, bidirectional regulator of antitumor immunity. Radiotherapy can induce systemic immune responses through immunogenic cell death and the abscopal effect. It can also mobilize suppressive cells and injure normal tissue. The clinical studies gathered here demonstrate meaningful benefit from radio-immunotherapy across liver, lung, cervical, and other cancers. The translational and mechanistic studies provide insights into which patients are most likely to benefit. They also examine how radiation should be delivered and how treatment-related toxicity can be anticipated. Notably, this Research Topic contains no contribution on radiation-based delivery platforms. These platforms include engineered nanoparticles, irradiated tumor cells, and tumor-derived microparticles or extracellular vesicles. This gap highlights an important direction for future research.

We thank all contributing authors for their excellent work. We thank the reviewers for their careful and constructive evaluations. We also thank the Frontiers editorial team for their support in assembling this Research Topic. We hope these articles stimulate further mechanistic and clinical study of how radiation and immunity intersect. Such work may help translate the combined potential of these two modalities into routine clinical benefit for patients.
